# Increased Mitochondrial Calcium Sensitivity and Abnormal Expression of Innate Immunity Genes Precede Dopaminergic Defects in *Pink1*-Deficient Mice

**DOI:** 10.1371/journal.pone.0016038

**Published:** 2011-01-13

**Authors:** Ravi S. Akundi, Zhenyu Huang, Joshua Eason, Jignesh D. Pandya, Lianteng Zhi, Wayne A. Cass, Patrick G. Sullivan, Hansruedi Büeler

**Affiliations:** 1 Department of Anatomy and Neurobiology, University of Kentucky College of Medicine, Lexington, Kentucky, United States of America; 2 Spinal Cord and Brain Injury Research Center, University of Kentucky College of Medicine, Lexington, Kentucky, United States of America; National Institutes of Health, United States of America

## Abstract

**Background:**

PTEN-induced kinase 1 (PINK1) is linked to recessive Parkinsonism (EOPD). *Pink1* deletion results in impaired dopamine (DA) release and decreased mitochondrial respiration in the striatum of mice. To reveal additional mechanisms of *Pink1*-related dopaminergic dysfunction, we studied Ca^2+^ vulnerability of purified brain mitochondria, DA levels and metabolism and whether signaling pathways implicated in Parkinson's disease (PD) display altered activity in the nigrostriatal system of *Pink1^−/−^* mice.

**Methods and Findings:**

Purified brain mitochondria of *Pink1^−/−^* mice showed impaired Ca^2+^ storage capacity, resulting in increased Ca^2+^ induced mitochondrial permeability transition (mPT) that was rescued by cyclosporine A. A subpopulation of neurons in the substantia nigra of *Pink1^−/−^* mice accumulated phospho-c-Jun, showing that Jun N-terminal kinase (JNK) activity is increased. *Pink1^−/−^* mice 6 months and older displayed reduced DA levels associated with increased DA turnover. Moreover, *Pink1^−/−^* mice had increased levels of IL-1β, IL-12 and IL-10 in the striatum after peripheral challenge with lipopolysaccharide (LPS), and *Pink1^−/−^* embryonic fibroblasts showed decreased basal and inflammatory cytokine-induced nuclear factor kappa-β (NF-κB) activity. Quantitative transcriptional profiling in the striatum revealed that *Pink1^−/−^* mice differentially express genes that (i) are upregulated in animals with experimentally induced dopaminergic lesions, (ii) regulate innate immune responses and/or apoptosis and (iii) promote axonal regeneration and sprouting.

**Conclusions:**

Increased mitochondrial Ca^2+^ sensitivity and JNK activity are early defects in *Pink1^−/−^* mice that precede reduced DA levels and abnormal DA homeostasis and may contribute to neuronal dysfunction in familial PD. Differential gene expression in the nigrostriatal system of *Pink1^−/−^* mice supports early dopaminergic dysfunction and shows that *Pink1* deletion causes aberrant expression of genes that regulate innate immune responses. While some differentially expressed genes may mitigate neurodegeneration, increased LPS-induced brain cytokine expression and impaired cytokine-induced NF-κB activation may predispose neurons of *Pink1^−/−^* mice to inflammation and injury-induced cell death.

## Introduction

Mutations in the *PARK6* gene encoding PINK1 are the second most frequent cause for EOPD [1], [2]. PINK1 is a ubiquitously expressed serine/threonine kinase with a mitochondrial targeting sequence that directs import of PINK1 into mitochondria [2], [3], [4]. In cultured cells, PINK1 protects against oxidative stress-induced cytochrome c release and apoptosis through phosphorylation of the mitochondrial chaperone TRAP1 [5]. *Pink1*-deficient *Drosophila* displayed mitochondrial degeneration associated with apoptotic muscle degeneration and DA neuron loss, which could be rescued by overexpression of Parkin [6], [7], [8], [9]. Work in cultured mammalian cells has shown that PINK1 directly phosphorylates Parkin [10] and that PINK1 is required for recruitment of Parkin to mitochondria with impaired membrane potential [11], [12]. In turn, Parkin promotes the degradation of functionally impaired mitochondria through ubiquitination-dependent autophagy [11], [12], [13]. Thus, PINK1 and Parkin cooperate in mitochondrial quality control by selectively promoting the degradation of dysfunctional mitochondria [14], [15]. In contrast to the severe mitochondrial defects and degenerative phenotypes of *Pink1*-deficient flies, mice lacking *Pink1* showed normal numbers and morphology of mitochondria and failed to develop DA neuron loss [16], [17]. Instead, they manifested milder defects, including impaired evoked DA release and mitochondrial respiration in the striatum [16], [17], [18]. The reason for the different phenotypes in mice and *Drosophila* is not clear, but it is conceivable that mice have a greater capacity to compensate for *Pink1* deficiency than flies. Such compensatory changes may include enhanced autophagy [19], [20] or increased mitochondrial biogenesis [21]. Alternatively, *Pink1*-defcient mice may compensate through changes in the expression of genes that protect against the effects of *Pink1* ablation *in vivo*, possibly downstream of mitochondrial dysfunction. It has also not been studied whether *Pink1* deficiency affects the activity of cell death pathways implicated in PD, such as the MAP kinase pathway [22]. To further study the consequences of *Pink1* gene deletion in mice and its effects on gene expression in the nigrostriatal system, we have generated and analyzed a new line of *Pink1*-deficient mice. Here we demonstrate that mitochondria from the brain of *Pink1*-deficient mice undergo Ca^2+^-induced permeability transition at a lower threshold and that pro-apoptotic Jun N-terminal kinase (JNK) signaling is increased in the substantia nigra of *Pink1^−/−^* mice. Importantly, DA levels are reduced in *Pink1^−/−^* mice six months and older, which is associated with increased DA turnover. We further show that ablation of *Pink1* results in reduced cytokine-induced NF-κB activity in *Pink1^−/−^* embryonic fibroblasts and increased levels of IL-1β, IL-10 and IL-12 in the striatum of *Pink1^−/−^* mice challenged with a low dose of LPS. Quantitative transcriptional profiling revealed that genes known to become activated after dopaminergic lesions were upregulated in the striatum of two month-old *Pink1^−/−^* mice, indicative of early dopaminergic dysfunction. Interestingly, several genes that participate in axonal regeneration and/or inhibit innate immune responses were overexpressed, while certain pro-inflammatory and apoptotic genes associated with neurodegeneration showed lower expression in *Pink1^−/−^* mice. This suggests that *Pink1^−/−^* mice may mitigate neurodegeneration by adapting the expression of a set of genes towards increased neuroprotection. Taken together, our results show that *Pink1* ablation enhances the sensitivity to Ca^2+^-induced mitochondrial permeability transition, triggers pro-apoptotic JNK signaling and causes a decline in striatal DA levels associated with increased DA turnover. Transcriptional profiling data suggest that *Pink1* deletion may cause neuroinflammation and axonal damage, which are compensated for by specific changes in gene expression. While these changes may in part prevent neurodegeneration in *Pink1^−/−^* mice, increased expression of cytokines in the striatum in response to peripheral inflammation may cause enhanced sensitivity to neuroinflammation, oxidative stress and brain injury. Further characterization of the role of these mechanisms in neuroprotection or neuronal loss will lead to better animal models for recessive Parkinsonism, as well as the identification of pathways that may be exploited as future targets for PD therapy.

## Results

### Generation and molecular characterization of *Pink1*-deficient mice

**Figure 1:**
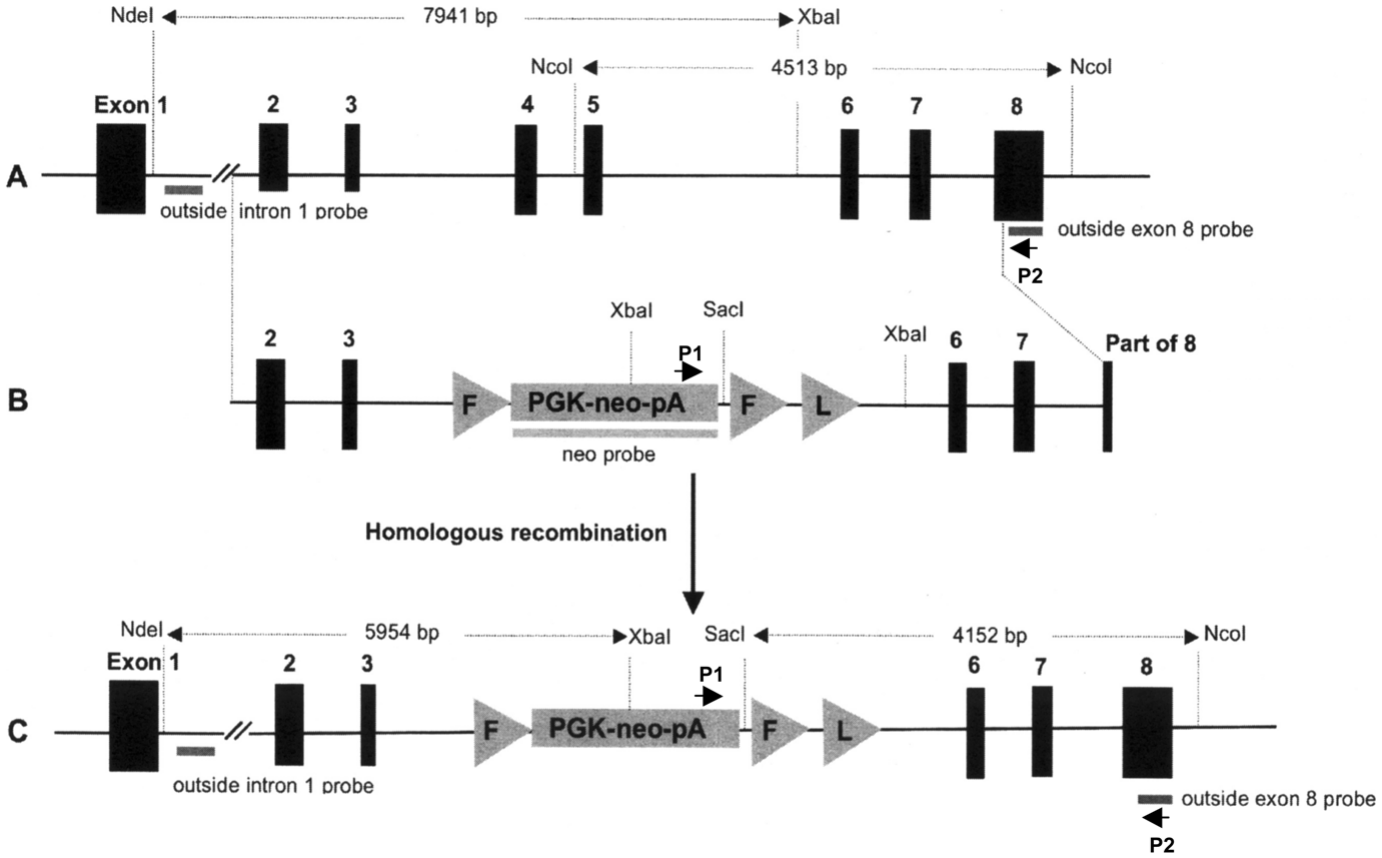
Inactivation of the mouse *Pink1* locus by gene targeting in ES cells. (A) Mouse *Pink1* gene structure, (B) targeting vector and (C) mutated *Pink1* gene lacking exons 4 and 5 after homologous recombination with the targeting vector. The PINK1 kinase domain is encoded within exons 2–8, with exons 4 and 5 specifying amino acids 257–374. Active site Asp362 and at least 15 familial PD-associated *Pink1* mutations cluster in exons 4 and 5 [143].

**Figure 2:**
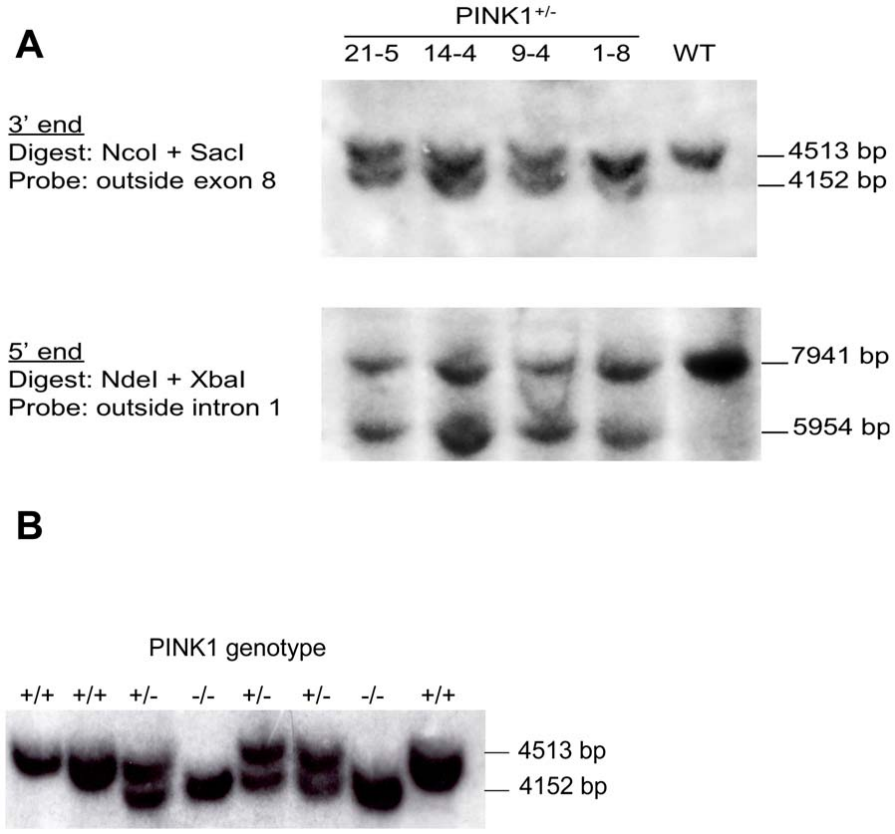
Southern blot analysis of ES cell colonies and F2 generation mice. (A) Genomic DNA from PCR-positive ES cell clones was double-digested with NcoI/SacI or NdeI/XbaI and hybridized with the indicated probes. The location of the two probes and expected sizes of the various DNA fragments for wildtype (WT) and *Pink1^+/−^* ES cells are shown in Figure 1. (B) Tail DNA from offspring of *Pink1^+/−^* breeder pairs was digested with NcoI and SacI and analyzed by Southern blot using “outside exon 8” probe. The 4513 bp band indicates the wildtype *Pink1* allele and the 4152 bp band is diagnostic for the mutated *Pink1* allele. Location of the probe and restriction enzyme cleavage sites are shown in Figure 1.

**Figure 3:**
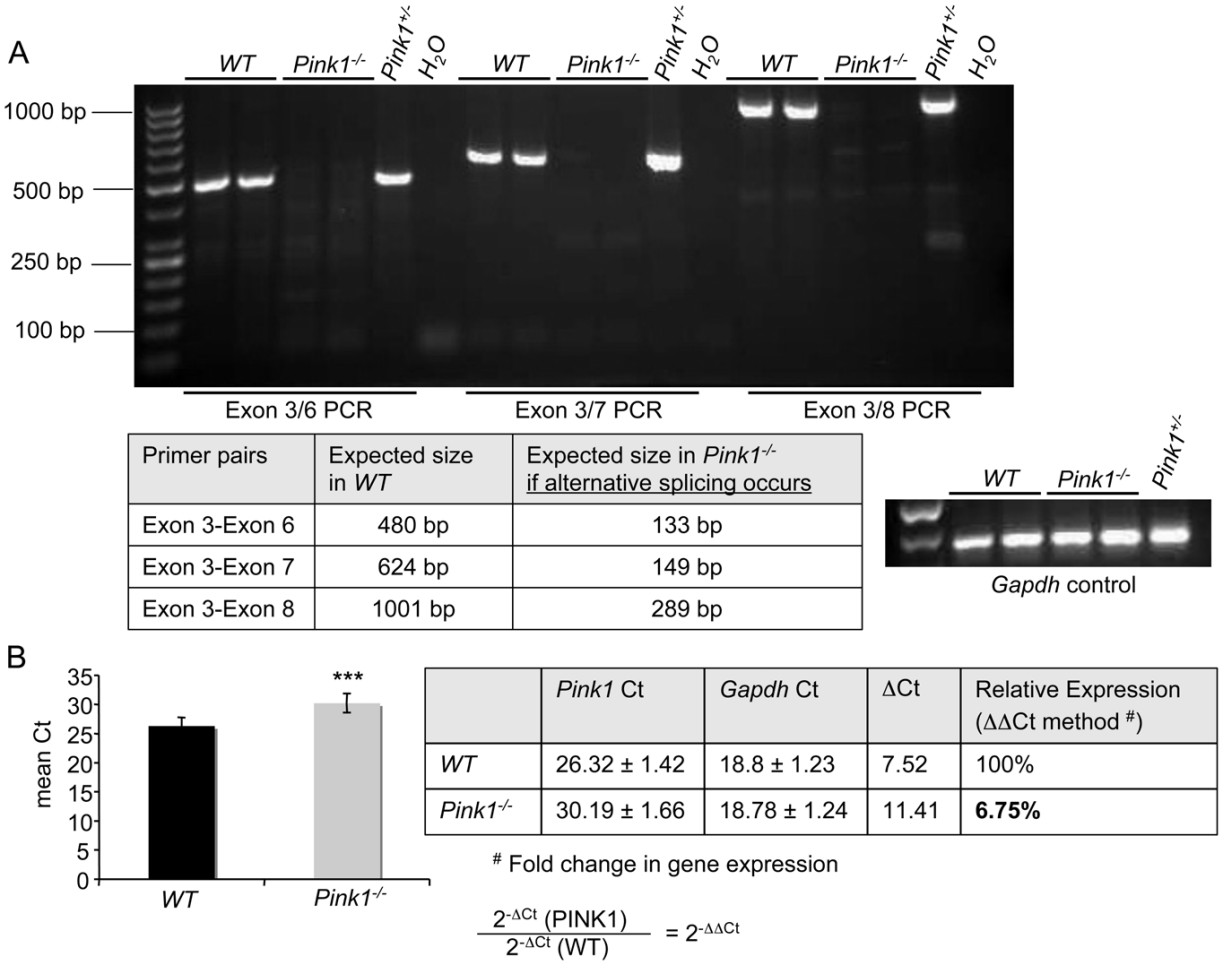
Analysis and quantification of *Pink1* mRNA expression. Total RNA isolated from the brain of mice was converted to cDNA. (A) PCR with a forward primer located in exon 3 and reverse primers located in exon 6, 7 or 8, generating expected PCR products of 480, 624 and 1001 bp for the *Pink1* wildtype allele in wildtype and *Pink1^+/−^* samples. In contrast, PCR products of 133, 149 and 289 that would arise if exon 3 were directly spliced to exon 6, 7, or 8 in transcripts derived from the mutant *Pink1* allele were absent in both *Pink1^+/−^* and *Pink1^−/−^* samples. This shows that alternatively spliced transcripts are *not* generated from the disrupted *Pink1* allele. (B) Quantitative real-time PCR with primers located in exon 1 (forward) and exon 3 (reverse) showing that *Pink1^−/−^* mice express at most 6.8% of a truncated *Pink1* mRNA encompassing exons 1–3, compared to wildtype mice. However, such a truncated mRNA would not give rise to any PINK1 protein with kinase activity (see main text).

Homologous recombination in mouse ES cells transfected with the targeting vector (construction described in the Methods) led to the replacement of *Pink1* exons 4 and 5 with a phosphoglycerate kinase (PGK) promoter-driven neomycin phosphotransferase (neo) expression cassette and the deletion of essential portions of the PINK1 kinase domain (Figure 1). About 2% of the G418-resistant ES colonies harbored a heterozygous deletion of the *Pink1* gene (Fig. 1C). These gene-targeted clones were identified by PCR screening with primers P1 and P2 (Fig. 1) and subsequently confirmed by Southern blot analysis using probes homologous to sequences 5′ and 3′ of the targeting vector (Fig. 2A). Internal probes were used to rule out additional random insertions of the targeting vector in the genome of the targeted clones (data not shown). Injection of two gene-targeted ES cell clones yielded highly chimeric mice that transmitted the *Pink1* gene deletion to their offspring. Genomic Southern blot analysis of tail DNA from the F2 generation demonstrated the presence of all three genotypes (Fig. 2B). Next, we verified that *Pink1^−/−^* mice lacked functional *Pink1* mRNA. First, expression of exon 4-containing *PINK1* transcripts was quantified by real-time reverse transcription PCR with primers located in *Pink1* exon 3 and exon 4, the latter of which is deleted in the mutant *Pink1* allele. *Pink1* mRNA expression was normalized to 18S rRNA levels in the same samples. The ratio of *Pink1* mRNA to 18S rRNA was 0.82±0.12 for wildtype mice (n = 3). A single *Pink1^+/−^* mouse showed a value of 0.52 (63% of wildtype), while *Pink1^−/−^* mice yielded an average signal of 0±0 (n = 6). This shows that no exon 4-containing *Pink1* transcripts were detected in *Pink1^−/−^* mice. Second, to analyze for the possibility of alternatively spliced transcripts originating from the mutated *Pink1* allele, we carried out PCR with forward primers located in exon 3 and reverse primers located in exon 6, 7 or 8. These primer pairs are expected to generate PCR products of specific lengths for the *Pink1* wildtype allele, and correspondingly shorter PCR products if alternatively spliced mutant *Pink1* transcripts are present in cells (Fig. 3A). All expected bands for full-length *Pink1* transcripts were detected in wildtype and heterozygous mutant (*Pink1^+/−^*) animals. In contrast, the *Pink1^−/−^* mice failed to produce the same bands (as expected) as well as any bands indicative of alternative splicing (Fig. 3A). These results show that alternative splicing (exon 4/5 skipping) does not occur in *Pink1^−/−^* mice. Third, we carried out real-time PCR with primers located in exon 1 and 3. As shown in Fig. 3B, the levels of transcripts encompassing exons 1–3 in the brain of *Pink1^−/−^* mice were reduced to 6.8% of wildtype. Using an N-terminal antibody against PINK1 [23], we also examined whether a low amount (less than 6.8%) of a truncated form of PINK1 might still be expressed in *Pink1^−/−^* mice. We used total brain, heart and embryonic fibroblast extracts from wildtype and *Pink1^−/−^* mice for Western blot analysis. However, although the antibody was shown to detect PINK1 in transfected cells [23], it was not sensitive enough to reveal the much lower levels of endogenous PINK1, because no specific bands of either 66 kDa (mitochondrial PINK1) or 55 kDa (cytosolic PINK1) were observed in any of the wildtype tissues and cells tested (data not shown). The lack of suitable antibodies to detect endogenous Pink1 was also reported by others [17]. We conclude that the targeted *Pink1* gene deletion prevented the generation of normal and aberrantly spliced *Pink1* transcripts. Furthermore, if *Pink1^−/−^* mice express an N-terminal portion of PINK1 protein, its levels are very low (less than 6.8% of normal amounts) and it lacks any kinase activity. Therefore, the mice described in this work are functionally PINK1-deficient.

### Brain mitochondria from *Pink1*-deficient mice display reduced threshold for calcium-induced permeability transition pore (PTP) opening

**Figure 4:**
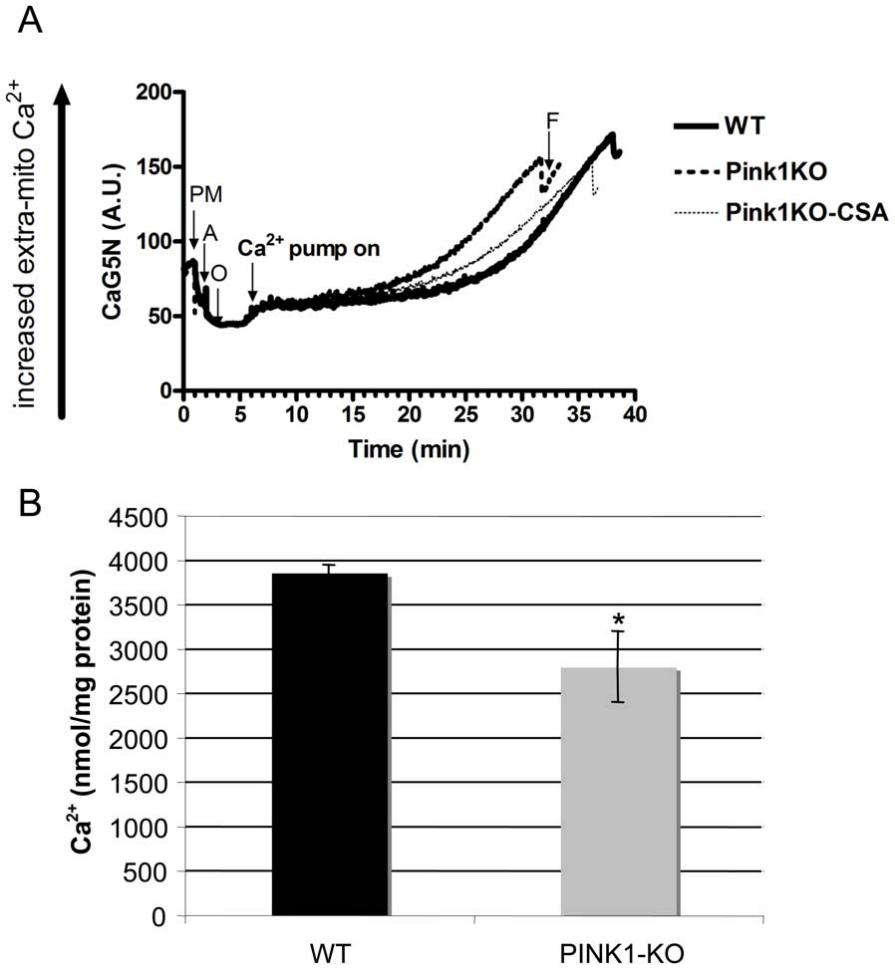
*Pink1^−/−^* brain mitochondria have significantly lower Ca^2+^ load capacity. Brain mitochondria were isolated from 2 month-old male wildtype (WT) and *Pink1^−/−^* (PINK1-KO) mice to measure calcium load capacity. Mitochondrial protein (200 µg) from each genotype was incubated in a 2-ml reaction mixture containing 125 mM KCl buffer (37°C) along with fluorescence indicators: 100 nM Calcium Green 5-N (for measurement of extra-mitochondrial calcium concentration; excitation 506 nm, emission 532 nm) and 100 nM TMRE (for measurement of membrane potential; excitation 550 nm, emission 575 nm). Mitochondrial bioenergetic coupling was assessed by following changes in membrane potential (TMRE fluorescence, data not shown) following the addition of pyruvate + malate (PM), ADP (A) and oligomycin (O) at 1, 2 and 3 min respectively. Calcium infusion began at 5 min (rate of 160 nmol/mg protein/min) and changes in extra-mitochondrial Ca^2+^ were assessed (CaG5N fluorescence). In Panel A, representative traces are shown to depict the calcium uptake and storage capacity before the opening of the mitochondrial permeability transition (mPT) pore. Mitochondria from PINK1-KO mice had decreased calcium load capacity, indicated by the earlier onset of mPT (sharp rise in extra-mitochondrial Ca^2+^) as compared to WT mice. Calcium load capacity in PINK-KO mice was increased by incubating mitochondria with cyclosporine A (CSA, 1 µM), a specific inhibitor of the PTP, showing that the reduced calcium load capacity was due to enhanced mPT. In Panel B, quantitative measurements of maximal calcium load capacity before mPT for mitochondria isolated from WT and PINK1-KO mice are shown and expressed as nmol Ca^2+^ infused/mg protein. The graph in panel B illustrates a significant loss of calcium load capacity in PINK1-KO mice (*P<0.05, n = 4 mice per genotype).

Recently, it has been shown that mitochondria of *Pink1*-deficient neurons accumulate higher basal levels of Ca^2+^ in the matrix (due to reduced calcium efflux capacity) and display reduced mitochondrial Ca^2+^ storage capacity associated with Ca^2+^ overload [24]. To study whether mitochondria from the brain of *Pink1*-deficient mice show increased sensitivity to Ca^2+^, we exposed preparations of Ficoll-purified whole brain mitochondria from two month-old *Pink1*-deficient mice and wildtype controls to increasing concentrations of Ca^2+^, and measured PTP opening using the Ca^2+^-sensitive fluorescent dye CaG5N and TMRE to monitor changes in mitochondrial membrane potential (ΔΨ_M_) [25]. *Pink1*-deficient brain mitochondria displayed a significant reduction in Ca^2+^ buffering capacity, which could be ameliorated by the addition of the PTP inhibitor cyclosporine A (CsA) (Fig. 4A). As a consequence mitochondria from *Pink1^−/−^* mice underwent permeability transition (mPT) at significantly lower concentrations of Ca ^2+^ compared to those of wildtype mice (Fig. 4B). Because CaG5N is an indicator of the *extra*-mitochondrial calcium concentration, the results cannot be attributed to differences in the dye loading capacity between wildtype and *Pink1^−/−^* mitochondria. We also measured mitochondrial production of reactive oxygen species (ROS) [25] but found no difference in ROS production between mitochondria purified from *Pink1*-deficient and wildtype brain (data not shown), consistent with previous results [18].

### Accumulation of phosphorylated c-Jun in the substantia nigra of *Pink1^−/−^* mice

**Figure 5:**
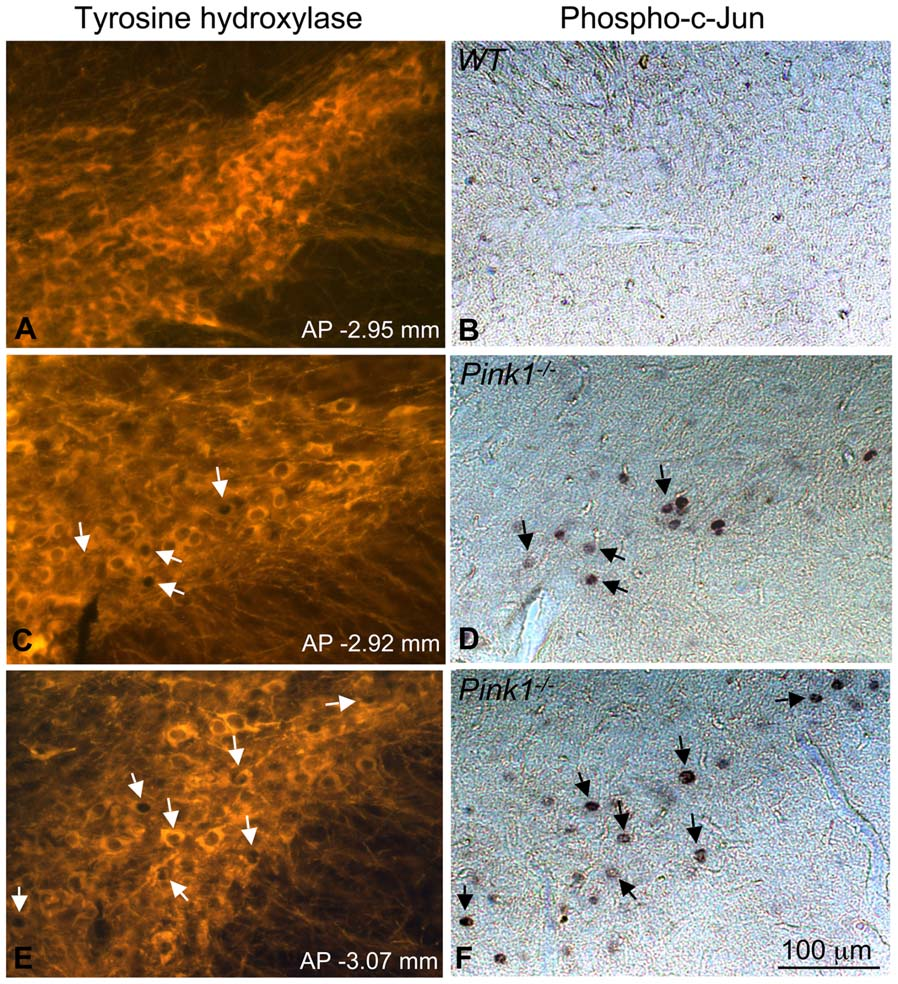
Accumulation of phospho-c-Jun in the substantia nigra of *Pink1^−/−^* mice. Cryosections of the substantia nigra from wildtype (A–B) and *Pink1^−/−^* mice (C–F) were stained with antibodies against phospho-c-Jun (nickel-DAB staining) and subsequently TH (fluorescent) as described in the Methods. Arrows in panels C–F point to cells expressing nuclear phospho-c-Jun that is surrounded by TH-positive cytosol, suggesting that these cells are dopaminergic neurons. AP coordinates of sections according to the mouse stereotaxic atlas (Franklin and Paxinos, The Mouse Brain in Stereotaxic Coordinates, Third Edition 2007) are indicated to demonstrate that phospho-c-Jun was expressed in distantly spaced sections of *Pink1^−/−^* mice (C–F), including sections anatomically matched to wildtype control mice (compare A–B and C–D). All three *Pink1^−/−^* mice but none of the wildtype mice showed expression of phospho-c-Jun in a subpopulation of TH-positive neurons.

JNK is a member of the MAP kinase family and has been implicated in neuronal cell death in a variety of circumstances including PD pathogenesis [22]. JNK is activated by oxidative and other types of stress and in turn phosphorylates c-Jun, its major substrate. In *Drosophila*, Parkin negatively regulates JNK activity [26]. To assess JNK activity in the nigrostriatal system of mice, we stained wildtype and *Pink1^−/−^* brain sections containing the substantia nigra with an antibody to phosphorylated c-Jun using the nickel-enhanced DAB method. Interestingly, we found that phospho-c-Jun accumulated in the substantia nigra of *Pink1^−/−^* but not wildtype mice (Fig. 5). At least a proportion of the phospho-c-Jun signals are most likely within nuclei of dopaminergic neurons, as they are surrounded by cytosol positive for tyrosine hydroxylase (TH) (arrows in Fig. 5C–F). To verify that phospho-c-Jun was expressed in dopaminergic neurons we carried out double labeling of phospho-c-Jun and TH using fluorescent secondary antibodies for analysis by confocal microcopy. However, phospho-c-Jun was not detected by fluorescent immunohistochemistry, suggesting that its expression was very weak (see Discussion). Nonetheless, expression of phosphorylated c-Jun was specific for *Pink1*-deficient mice, as all three *Pink1* knockout mice showed phospho-c-Jun expression while none of the wildtype mice did. These results show that increased JNK activation occurs in the substantia nigra of *Pink1*-deficient mice and may play a role in *Pink1*-related Parkinsonism.

### Decreased dopamine levels associated with increased dopamine turnover in *Pink1^−/−^* mice aged six months and older

**Figure 6:**
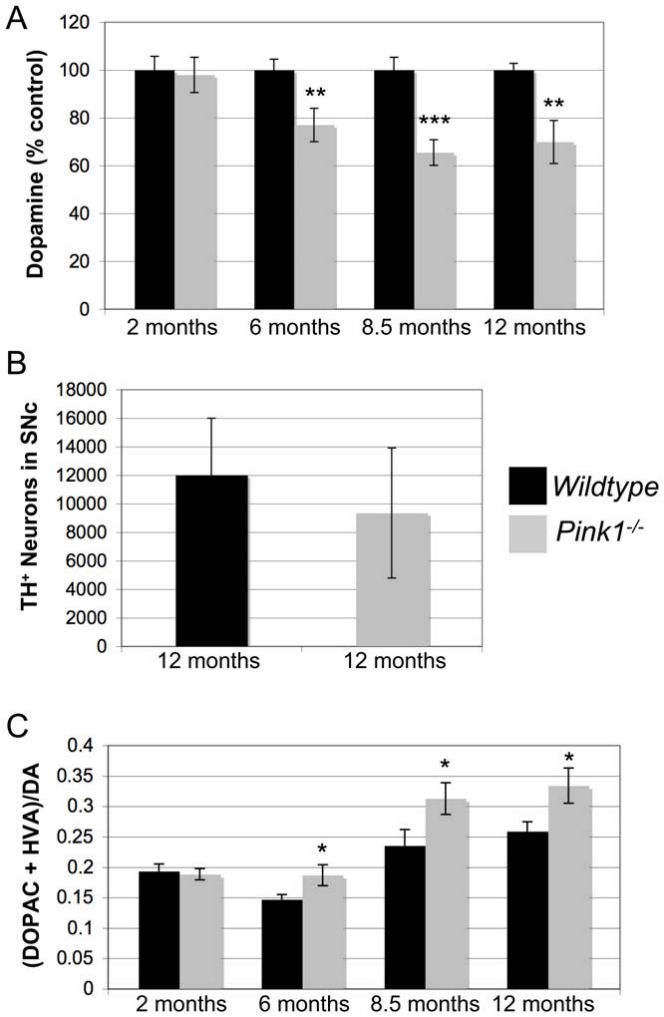
Dopamine levels, dopamine turnover and dopamine neuron counts. (A) Decreased DA levels in the striatum of *Pink1^−/−^* mice aged 6 months and older. (B) Normal counts of dopaminergic neurons in the substantia nigra pars compacta (SNc) of 1-year old *Pink1^−/−^* mice. (C) Increased DA turnover in *Pink1^−/−^* mice. Eight mice per genotype were used for catecholamine analysis (A and C). Five wildtype and six *Pink1^−/−^* mice were used to determine nigral DA neuron numbers by unbiased stereology (B). * *P*<0.05, ** *P*<0.01, *** *P*<0.001.

To study whether the targeted *Pink1* mutation affected dopaminergic parameters, we measured the levels of DA in the striatum by HPLC in mice between 2 and 12 months of age. *Pink1^−/−^* mice aged 6 months and older had significantly lower DA levels in the striatum than their wildtype controls (Fig. 6A). However, stereological quantification of DA neuron numbers in 1-year old *Pink1^−/−^* and wildtype mice showed no significant difference, although the average number was 22% lower in *Pink1^−/−^* mice (Fig. 6B). This suggested that dopaminergic neurons of *Pink1^−/−^* mice synthesize less dopamine and/or that dopamine metabolism is increased. In support of this, we show, for the first time, that DA turnover is increased in *Pink1^−/−^* mice at those ages where DA levels are lower (Fig. 6C). These results confirm that *Pink1* deficiency can cause lower DA levels [17] and further suggest that increased DA turnover may be involved in this defect.

### Loss of *Pink1* activates expression of stress-inducible transcription factor genes

To gain additional insights into mechanisms promoting dopaminergic dysfunction in response to *Pink1* ablation, we compared striatal gene expression profiles between two month-old wildtype and *Pink1^−/−^* mice. We focused on cAMP/Ca^2+^-regulated genes and genes of the Akt/protein kinase B (Akt/PKB) and nuclear factor kappa-β (NF-κB) pathways, because abnormalities in these pathways have been implicated in PD [27], [28], [29]. We found that several of the upregulated genes encoded stress-inducible transcription factors of the ATF and AP1 families, including activating transcription factor 3 (ATF-3), c-fos, FosB, JunB and Egr-2 (Table 1). ATF3 is induced by multiple signals, including inflammatory cytokines, DNA-damaging agents and physiological stresses [30], [31]. Interestingly, increased striatal expression of Fos-related antigens and JunB has been observed following neuronal injury and degeneration in the DA system [32], [33]. Moreover, striatal c-fos expression is regulated by DA [34] and, in the DA-depleted striatum, may be induced via compensatory super-sensitivity of DA receptors [35], [36]. Likewise, expression of Egr-2 is regulated in a D1 and D2 DA receptor-dependent fashion [37], [38].

**Table 1:** Genes with significantly altered expression in the striatum of *Pink1^−/−^* mice.

Gene	Description	Threshold Cycles		Expression relative to HKG		Expression	*P*	N
		Ct *WT*	Ct *Pink1^−/−^*	2^−(AVG ΔCt)^ *WT*	2^−(AVG ΔCt)^ *Pink1^−/−^*	*Pink1^−/−^/WT*		
***Upregulated Genes***								
Fos	FBJ osteosarcoma oncogene	29.29±1.40	27.36±1.62	1.81×10^−2^	5.06×10^−2^	2.80	0.031	5
Tnfrsf10b	TRAIL receptor 2	34.41±0.56	33.5±0.25	3.74×10^−4^	6.46×10^−4^	1.72	0.048	4
Nfkbia	NF-κB inhibitor-α	27.71±0.54	26.76±0.30	4.35×10^−2^	6.60×10^−2^	1.52	0.0002	5
Fosb	FBJ osteosarcoma oncogene B	30.73±1.31	28.62±0.97	2.65×10^−3^	8.24×10^−3^	3.12	0.019	4
Cyr61	Cysteine-rich protein 61	30.15±0.40	28.17±1.10	3.97×10^−3^	1.12×10^−2^	2.83	0.018	4
JunB	JunB oncogene	32.46±1.23	30.5±0.56	7.99×10^−4^	2.24×10^−3^	2.80	0.005	4
Egr2	Early growth response 2	28.51±1.17	26.6±1.06	1.24×10^−2^	3.34×10^−2^	2.68	0.049	4
Atf3	Activating transcription factor3	33.2±0.30	31.69±0.91	4.78×10^−4^	9.82×10^−4^	2.05	0.027	4
Dusp1	Map kinase phosphatase-1	26.6±0.28	25.12±0.61	4.64×10^−2^	9.34×10^−2^	2.01	0.0005	4
Areg	Amphiregulin	34.1±0.62	32.76±0.75	2.56×10^−4^	4.68×10^−4^	1.83	0.027	4
***Down-regulated Genes***								
Tnfrsf1b	TNF receptor 2	31.41±0.47	32.21±0.73	3.0×10^−3^	1.58×10^−3^	0.53	0.01	4
Tnfrsf1a	TNF receptor 1	32.01±0.56	32.49±0.63	1.97×10^−3^	1.30×10^−3^	0.65	0.022	4
Pcaf	p300/CBP-associated factor	26.56±0.52	27.01±0.37	8.72×10^−2^	5.76×10^−2^	0.65	0.035	4
Gsk3b	Glycogen synthase kinase-3β	25.38±0.71	25.67±0.76	2.55×10^−1^	1.70×10^−1^	0.66	0.01	4

Gene expression was analyzed using NF-κB, PI3 kinase/Akt and cAMP/Ca^2+^ signaling PCR Arrays (SA Biosciences) as described in the Methods. Ct values (mean ± SD) for individual genes are indicated for wildtype *(WT)* and *Pink1^−/−^* mice. In addition, expression of each gene relative to the housekeeping genes (HKG) is indicated for WT and *Pink1^−/−^* mice and was used to calculate the fold change in gene expression *(Pink1^−/−^/WT).* All PCR arrays contain five HKG (β-glucuronidase, hypoxanthine guanine phosphoribosyl transferase, heat shock protein 90-alpha, glyceraldehyde-3-phosphate dehydrogenase, and β-actin), to which the expression of the genes of interests is normalized. None of the HKG was differentially expressed between *WT* and *Pink1^−/−^* mice. Data were evaluated and calculated with the ΔΔCt method using the RT^2^ Profiler PCR Array Data Analysis software and resources available online (http://sabiosciences.com/pcr/arrayanalysis.php). The p values were calculated based on a Student's t-test of the replicate 2^−(AVG ΔCt)^ values for each gene in the *WT* and *Pink1^−/−^* groups. All genes with a p<0.05 that were upregulated or down-regulated by at least a factor of 1.5 are shown. N is the number of data points available for a given gene for *both* genotypes after running five arrays (five mice per genotype were analyzed). Occasionally, a well (gene) yielded no signal at all for reasons that are unrelated to actual lack of expression. For example, if a specific gene became detectable at PCR cycle number 25 in four mice but showed no expression in the fifth mouse of the same genotype, we concluded that this must be an experimental/technical error rather than actual lack of expression and omitted the corresponding data. However, we still used five data points for the other genotype if available. For a description of the function of the listed genes in innate immunity, MAPK signaling and/or their involvement and regulation in PD and models of PD, see the main text and references therein.

### Ablation of *PINK1* increases expression of Cyr61 and Amphiregulin

We also found significantly increased expression of Cyr61 and Amphiregulin in the striatum of *Pink1*-deficient mice (Table 1). Cyr61 is an immediate early gene induced downstream of JNK activation that has been linked to neurodegeneration [39]. Cyr61 transcription is negatively regulated by the forkhead transcription factor FOXO3a [40], which has been shown to activate the *Pink1* gene under conditions of growth factor deprivation [41]. Amphiregulin is a mitogen for adult neural stem cells [42] and acts as an autocrine survival factor for sensory neurons where it promotes axonal outgrowth [43].

### Altered expression of genes that regulate innate immunity and MAP kinase signaling in the striatum of *Pink1*-deficient mice

*Pink1^−/−^* mice displayed altered expression of many genes that regulate innate immune responses and the MAPK pathway (Table 1). For example, the inhibitor-α of NF-κB (IκB-α), MAPK phosphatase-1 (MKP-1/Dusp-1) and the receptor for tumor necrosis factor-related apoptosis inducing ligand (TRAIL-R2) were all upregulated in *Pink1^−/−^* striatum. IkB-α provides a negative feedback regulation for NF-κB signaling [44], [45]. MKP-1 inactivates p38 MAPK and JNK through dephosphorylation and via NF-κB regulation [46] and functions as an important negative regulator of innate and adaptive immune responses [46], [47], [48]. TRAIL-R2 is induced by a variety of stress factors and its activation involves both NF-κB and p53 dependent pathways [49], [50]. Four genes were expressed at lower levels in the striatum of *Pink1^−/−^* mice (Table 1). These were p300/CREB-binding protein associated factor (p300/CBP-associated factor, PCAF), tumor necrosis factor receptor-1α (TNF-R1), tumor necrosis factor receptor-1β (TNF-R2), and glycogen synthase kinase-3 beta (GSK-3β). The histone acetyltransferase PCAF functions as a transcriptional co-activator of a subset of NF-κB regulated genes [51], including cyclooxygenase-2 (COX-2) [52]. However, PCAF is also required for post-activation shut-off of NF-κB gene transcription through acetylation of p65 [53]. TNF receptors mediate numerous functions in cells that range from inflammation, apoptosis and cell survival [54], [55], [56]. TNF-R engagement activates NF-κB signaling and the MAPK pathway and thus often results in tissue inflammation [56]. GSK-3β has been linked to neurodegeneration and the neuropathology of PD [57], [58], [59], [60]. Taken together, these results show that several key regulators and effectors of the TNF-R, NF-κB and MAPK signaling pathways are abnormally expressed in the striatum of *Pink1*-deficient mice.

### Basal and inflammatory cytokine-induced NF-κB activity is reduced in *PINK1*-deficient mouse embryonic fibroblasts (MEF)

**Figure 7:**
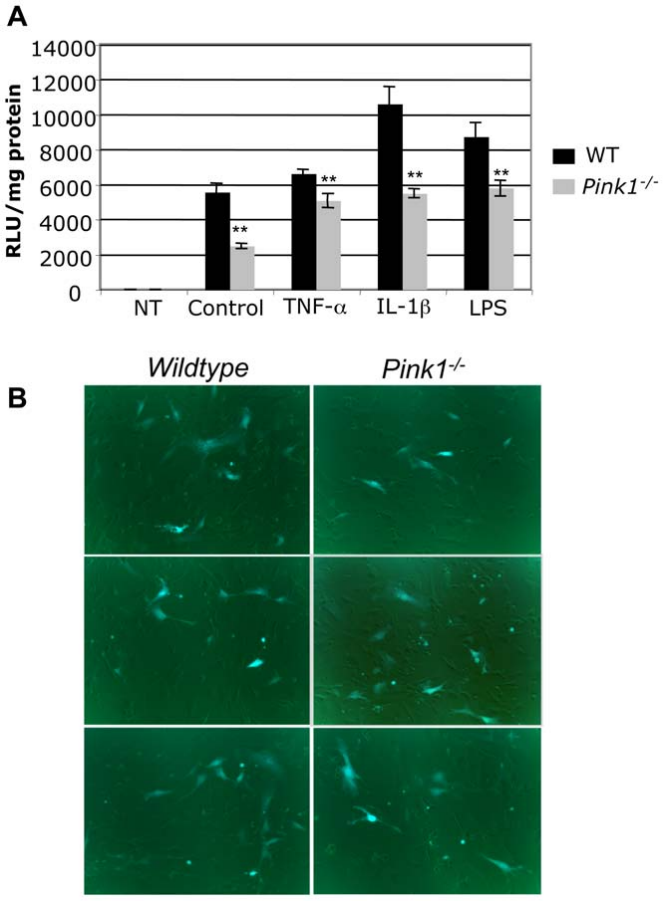
Basal and inflammatory signal-induced NF-κB activity is reduced in *Pink1^−/−^* embryonic fibroblasts. Wildtype and *Pink1^−/−^* MEF were transfected with plasmid pNF-κB-luc (Clontech). Twenty-four hours after transfection the cells were incubated for 8 hours with 30 ng/ml TNF-α, 10 ng/ml IL-1β, 100 ng/ml LPS or remained untreated (control) and luciferase activity was measured as described in the Methods. (A) NF-κB-dependent luciferase activity, expressed as relative light units (RLU) per mg protein. Data represent pooled values from two independent experiments with similar results. In each experiment luciferase activity was measured in five wells per condition. Non-transfected cells (NT) showed no luciferase activity. (B) Wildtype and *Pink1^−/−^* fibroblasts were transfected with the same plasmid/lipofectamine mixture to ensure equal transfection efficiency, which was confirmed to be the case with an EGFP expression plasmid as described in the Methods. ** *P*<0.01.

The transcriptional profiling results indicated abnormalities in TNF-R and NF-κB signaling in the striatum of *Pink1*-deficient mice. The inflammatory cytokines tumor necrosis factor-α (TNF-α) and interleukin 1β (IL-1β) are potent inducers of NF-κB signaling [61], [62]. In addition bacterial LPS activates the NF-κB pathway via Toll-like receptor 4 (TLR-4) and TNF-α expression. To study basal and inflammatory cytokine-induced NF-κB activity, we transfected primary MEF from *Pink1*-deficient and wildtype mice with an NF-κB-luciferase reporter plasmid and measured luciferase activity before and after treatment of cells with TNF-α and IL-1β. Compared to wildtype MEF, fibroblasts derived from *Pink1^−/−^* mice showed significantly reduced NF-κB activity in both the basal state and after treatment with inflammatory cytokines (Fig. 7A). Likewise, LPS-induced NF-κB activity was decreased in *Pink1^−/−^* MEF (Fig. 7A). EGFP expression in control wells of wildtype and *Pink1^−/−^* MEF (Fig. 7B) was used to confirm comparable transfection efficiency. These results show that lack of *Pink1* impairs NF-κB activity and reduces inflammatory signal-dependent NF-κB pathway induction.

### *Pink1^−/−^* mice show increased striatal levels of IL-1β, IL-12 and IL-10 after treatment with LPS

**Figure 8:**
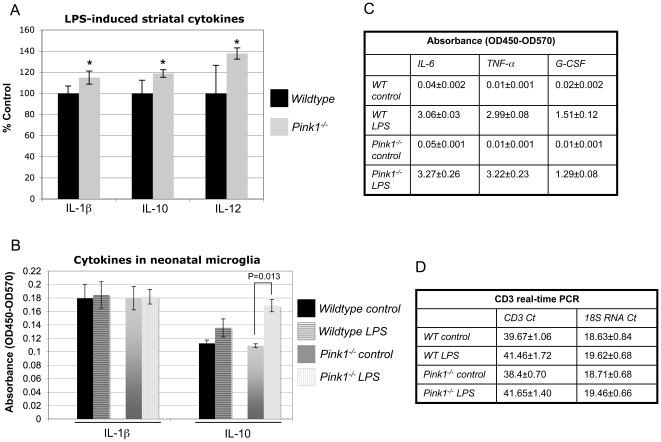
Cytokine expression in the striatum and isolated microglial cultures. (A) Wildtype and *Pink1^−/−^* mice (n = 4 mice/genotype) were injected ip with 0.33 µg LPS/g body weight. Cytokines in striatal homogenates (corresponding to 100 µg total protein) were measured eight hours later by ELISA as described in the Materials and Methods. * *P*<0.05, compared to wildtype mice. Basal cytokine levels, measured in a separate experiment, were not statistically different between wildtype and *Pink1^−/−^* mice. (B) Microglial cultures derived from the forebrain of neonatal wildtype and *Pink1^−/−^* mice were incubated with 100 ng/ml LPS for 24 hours in 24-well plates and the cytokines were measured with an ELISA as described in the Methods (n = 8 wells per condition). (C) Strong induction of IL-6, TNF-α and G-CSF in wildtype and *Pink1^−/−^* microglia cells demonstrates that the cells were capable of responding to the LPS stimulus. In panels B and C, background-corrected absorbance is plotted (OD450 minus OD570). (D) Real-time PCR expression analysis of CD3 mRNA (specific T cell marker) in the striatum of control and LPS-treated wildtype and *Pink1^−/−^* mice, showing that the T cell marker is barely detectable (Ct values of 39.67 and 38.46) and not increased by LPS treatment.

Given the increased expression of anti-inflammatory genes in the striatum and enhanced JNK activity in the substantia nigra of *Pink1^−/−^* mice, we were interested to test whether *Pink1^−/−^* mice showed abnormal expression of inflammatory and/or anti-inflammatory cytokines in the striatum. The relative striatal levels of twelve cytokines (IL-1α, IL-1β, IL-2, IL-4, IL-6, IL-10, IL-12, IL-17α, TNF-α, G-CSF, GM-CSF) were measured using an enzyme-linked immunosorbent assay (ELISA). We did not find a significant difference in the expression of these cytokines between wildtype and *Pink1^−/−^* mice (data not shown). However, after peripheral challenge with a low dose of LPS, *Pink1^−/−^* mice expressed higher levels of IL-1β, IL-12 and IL-10 in the striatum compared to wildtype mice (Fig. 8A). In addition, a tendency for increased expression of IL-2 (p = 0.053), IL-4 (p = 0.085) and TNF-α (p = 0.072) was observed. In the brain, microglia cells are the major source for LPS-induced cytokine production [63]. Therefore we studied cytokine production of cultured neonatal microglia in response to LPS. The levels of IL-10 were significantly higher after LPS in *Pink1^−/−^* but not wildtype microglia, suggesting that IL-10 secretion is potentiated in *Pink1^−/−^* microglia (Fig. 8B). This is in agreement with increased IL-10 levels in the striatum of *Pink1^−/−^* mice (Fig. 8A). However, IL-1β levels did not significantly increase in cultured microglia from either genotype (Fig. 8B) and IL-12 levels were too low to be detected with the ELISA (data not shown). In contrast, the expression of IL-6, TNF-α and G-CSF was dramatically induced after LPS in both genotypes, showing that the microglia cells were competent to respond to an inflammatory stimulus (Fig. 8C). As T cells can also synthesize cytokines, we quantified the expression of the T cell marker CD3 in the striatum by real-time PCR. T cells are not normally present in significant numbers in the brain. Consistent with this the expression of CD3 in the striatum of normal mice was very low, as evidenced by Ct values in the range of 39 (Fig. 8D). In addition, CD3 expression did not increase after LPS treatment. Thus, we believe that T cells are likely not involved in augmenting brain cytokine levels in LPS-treated *Pink1^−/−^* mice. This is consistent with a role for T cells in antigen-specific adaptive immune responses, which take longer to develop than eight hours between LPS injection and cytokine analysis in our experiment. Taken together, these results show that *Pink1^−/−^* mice display abnormal brain cytokine expression in response to peripheral LPS injection and suggest that *Pink1^−/−^* mice may be more susceptible to inflammation-induced DA neuron death [64], [65], [66], [67], [68].

## Discussion

### Ca^2+^-induced mitochondrial permeability transition is increased in the absence of *Pink1*

It has been shown that striatal mitochondria from *Pink1^−/−^* mice show impaired state 3 respiratory activities of complex I and II and that *Pink1^−/−^* cortical mitochondria are more sensitive to H_2_O_2_-induced defects, while the levels of anti-oxidant enzymes were normal [18]. Here, we show for the first time that isolated purified mitochondria from the brain of *Pink1^−/−^* mice display an enhanced vulnerability to Ca^2+^-induced mPT. Whereas cultured neurons of *Pink1^−/−^* mice were reported to be more sensitive to Ca^2+^-induced cell death due to Ca^2+^ overload [24], mitochondria of *Pink1*-deficient mice did not appear to be more sensitive to Ca^2+^ than mitochondria from wildtype mice [18]. This led to the conclusion that, independent of Ca^2+^ and the mitochondrial PTP, *Pink1^−/−^* mitochondria are more sensitive to stress [18]. However, in these experiments the swelling assay was used and only one concentration of Ca^2+^ was tested. Additionally, a differential effect of Ca^2+^ loading could have been missed due to low coupling of the mitochondrial preparations used in these experiments (RCR<5). In contrast, we incubated highly coupled mitochondria (RCR>10) with increasing concentrations of Ca^2+^, and measured mitochondrial Ca^2+^ uptake and the sudden Ca^2+^ release and loss of ΔΨ_M_ at the time of PTP opening using the Ca^2+^-sensitive dye CaG5N and TMRE to monitor ΔΨ_M_ [25]. Our results show that freshly isolated brain mitochondria lacking *Pink1* undergo mPT at lower Ca^2+^ concentrations than mitochondria from wildtype mice. Thus, it is likely that the facilitated Ca^2+^-induced mPT in *Pink1*-deficient mitochondria renders neurons in the brain more vulnerable to Ca^2+^-mediated death. Interestingly, we observed enhanced Ca^2+^-induced mitochondrial PTP opening with whole brain mitochondria, showing that this defect is not specific for the dopaminergic system. Because Ca^2+^ plays an important role in the physiology of all neurons, this raises the question of how *Pink1* mutations in humans lead to the selective loss of DA neurons. An unusual aspect of adult dopaminergic neurons is that they express unique L-type Ca^2+^ channels required for rhythmic pace-making and tonic DA release, and blocking Ca^2+^ influx through these channels has been shown to protect against toxin-induced dopaminergic system degeneration in animal models of PD [69], [70]. Therefore, the life-long reliance on L-type Ca^2+^ channels may render DA neurons particularly vulnerable to perturbations in mitochondrial Ca^2+^ buffering capacity. This hypothesis can be tested in future experiments with neurons derived from *Pink1*-deficient mice.

### Increased JNK activity in the substantia nigra of *Pink1^−/−^* mice

We found that phosphorylated c-Jun accumulates in the substantia nigra of *Pink1^−/−^* mice. Nuclear phospho-c-Jun was clearly surrounded by TH-positive cytosol in at least a proportion of the cells, suggesting that phospho-c-Jun is expressed in dopaminergic neurons. To further investigate this, we attempted colocalization of TH and phospho-c-Jun by confocal microscopy. However, phospho-c-Jun was not detectable with fluorescent secondary antibodies, while the highly sensitive nickel-enhanced DAB staining method was able to reveal phospho-c-Jun expression. We have observed that detection of at least one other protein, c-fos, is significantly more sensitive with the nickel-DAB method compared to fluorescent immunohistochemistry. Similar c-fos signals were obtained with a 50-fold higher dilution of the primary antibody (1∶15000) by the nickel-DAB method when compared to fluorescent detection (1∶300) (data not shown). As phospho-c-Jun was detected with 1∶300-diluted primary antibody only with the nickel-DAB method, we conclude that its expression is very weak. In the absence of confocal colocalization we cannot conclude with certainty that phospho-c-Jun is expressed within dopaminergic neurons of *Pink1^−/−^* mice, although we believe this to be likely based on the data presented in Figure 5. We have not studied whether the phospho-c-Jun positive neurons express other markers such as neurogenin [18] and dopamine and cAMP-regulated phosphoproteins (DARPP-32), a regulator of DA-induced signal transduction [71]. Increased JNK signaling has been shown to promote dopaminergic neuron death [22], [72]. Phospho-c-Jun has been detected in cytosolic granules adjacent to Lewy bodies in neurons in PD and dementia with Lewy bodies [73]. Moreover, genetic deletion of specific JNK isoforms prevented complex I inhibitor (MPTP)-mediated [74] and axotomy-induced [75] cell death of nigral dopaminergic neurons in animal models of PD. These results suggest that JNK may be activated downstream of mitochondrial and possibly axonal damage in *Pink1^−/−^* mice. In *Drosophila*, parkin deletion resulted in the activation of JNK in a small subgroup of dopaminergic neurons that underwent degeneration, suggesting that parkin negatively regulates JNK signaling [26]. Because PINK1 kinase activity is required for many Parkin functions and Parkin acts downstream of PINK1 [11], [14], [76], increased JNK signaling in *Pink1^−/−^* mice may in part be due to reduced Parkin activity. Taken together, these data implicate *Pink1* in the inhibition of JNK signaling and the mitigation of the effects of pro-apoptotic MAP kinase signaling.

### Abnormal dopamine levels and turnover in the striatum of *Pink1^−/−^* mice

Importantly, we show that *Pink1^−/−^* mice aged 6 months and older have significantly lower DA levels in the striatum than their wildtype controls. This is in contrast to another group that found DA levels to be normal in 8–9 month-old *Pink1^−/−^* mice [16]. However, reduced DA levels were found in *Pink1^−/−^* mice of independent origin at 9 and 22–24 months of age, although the dopaminergic neuron counts in the SNc were normal [17]. Our data are in agreement with the latter results. However, the *Pink1^−/−^* mice described here show a significant decline in DA levels at a much earlier age than previously reported [17]. Furthermore, we show for the first time that DA turnover is increased in *Pink1^−/−^* mice, providing a potential mechanism for the decline in DA in the absence of (significant) neuronal loss. It is well established that PD is characterized by increased DA turnover which occurs early in the disease [77]. Increased DA turnover is associated with elevated oxidative stress [78], which may exarcerbate dopaminergic dysfunction in the long-term. Thus, our studies suggest that abnormal DA homeostasis may precede and contribute to neuronal loss in *Pink1*-related Parkinsonism.

### Transcriptional changes in the striatum of young *Pink1^−/−^* mice indicate early dopaminergic dysfunction preceding dopamine loss

Quantitative striatal gene expression analyses showed that the genes displaying altered expression in *Pink1^−/−^* mice could be grouped into three categories. The first and largest category contained genes that regulate innate immune responses and/or apoptosis. Within this category, MKP-1, ATF3 and TRAIL-R2 were upregulated in *Pink1^−/−^* mice. MKP-1 attenuates JNK-dependent apoptosis [46] and is upregulated specifically in healthy but not degenerating neurons after axotomy, demonstrating that it participates in a neuronal stress response promoting survival [79]. MKP-1 also acts as a negative regulator of innate immune responses by suppressing the expression of pro-inflammatory cytokines, endotoxic shock and activation of both p38 MAPK and JNK [80], [81], [82]. Likewise, ATF3 and TRAIL-R2 are stress-inducible genes that reduce innate immune responses and tissue damage caused by chronic inflammation through their capacity to inhibit Toll-like receptor signaling [83], [84], [85], [86]. In contrast, the expression of GSK-3β and both TNF receptors, which mediate pro-inflammatory signals, was suppressed in *Pink1^−/−^* mice. Deficiency of TNF receptors inhibits microglial activation [87] and protects mice partially against MPTP-induced loss of dopaminergic terminals and DA decline [67]. The role of GSK-3β in innate immunity is complex and its effect on NF-κB function appears to depend on the cell type studied [88], [89], [90]. Reduced GSK-3β expression may be a protective adaptation to increased JNK signaling in *Pink1^−/−^* mice, because GSK-3β inhibition has been shown to block pro-apoptotic JNK [91]. On the other hand, GSK-3β also functions as a negative regulator of pro-inflammatory cytokine expression [92], [93] and decreased GSK-3β expression may render *Pink1^−/−^* mice more vulnerable to TNF-α, IL-1β and LPS-induced brain inflammation [88], [91], [94]. However, it remains to be seen whether decreased GSK-3β expression is associated with altered GSK-3β activity in the striatum of *Pink1^−/−^* mice. Overall, the genes identified in category 1 suggest that ablation of *Pink1* results in increased brain inflammation and MAP kinase pathway activation, possibly through heightened oxidative stress [95], [96], [97], [98]. In response, *Pink1^−/−^* mice appear to adapt the expression levels of critical genes controlling innate immune responses, perhaps to mitigate inflammation and inflammation-induced neuronal damage (see also below).

Category 2 contained immediate-early transcription factors, including c-fos, FosB and Egr2, which were among the most highly overexpressed in the striatum of *Pink1^−/−^* mice (Table 1). The same genes were upregulated in the striatum of animals with toxin or surgery-induced dopaminergic deficits, or chronic alterations of dopaminergic neurotransmission, which depended on D1 and D2 DA receptor signaling [35], [99], [100]. In addition, c-fos, FosB and Egr-2 were all upregulated by methamphetamine, which can cause long-lasting neurodegenerative effects that are at least in part due to activation of D1 DA receptors [37]. Collectively, these results suggest increased DA receptor signaling in the striatum of *Pink1*-deficient mice, possibly as compensation to reduced DA neurotransmission. Consistent with this, reduced KCl-evoked DA release was described in acute striatal slices from 2–3 month-old *Pink1^−/−^* mice previously [16]. Our results indicate that, in addition to evoked DA release, tonic DA release may also be affected in *Pink1^−/−^* mice, leading to lasting elevations in postsynaptic striatal expression of immediate early genes. In addition Cyr61 was upregulated in *Pink1^−/−^* mice. Cyr61 is an immediate-early gene induced downstream of JNK activation that has been implicated in neurodegeneration [39].

Several genes of category 3 are induced in response to axonal injury and have been shown to promote axonal outgrowth and sprouting. In particular, overexpression of JunB and amphiregulin in the striatum of *Pink1^−/−^* mice may be neuroprotective. JunB transgenic mice displayed significantly increased long-term survival of substantia nigra dopaminergic neurons after axotomy [101]. Amphiregulin is a mitogen for adult neural stem cells [42] and has been shown to promote axonal outgrowth [43]. Several groups described that ATF3 stimulates axonal outgrowth and sprouting after neuronal injury [102], [103], [104], [105]. Overexpression of ATF3 protected hippocampal neurons against excitotoxic cell death [106] and PC12 cells against mutant huntingtin-induced toxicity [107]. However, ATF3 has also been shown to promote cell death by mediating the apoptotic effects of p38 MAPK [31], and its expression preceded the death of spinal motor neurons and correlated with phosphorylation of c-Jun in a mouse model of familial amyotrophic laterals sclerosis [108]. Furthermore, it has been proposed that, in the nigrostriatal system, ATF3 and phospho-c-Jun participate in axotomy-induced neurodegeneration [109]. However, another study showed that ATF3 inhibits JNK-mediated neuron death through induction of Hsp27 expression and Akt activation [110]. Thus, an alternative interpretation is that ATF3 may be induced in response to increased JNK signaling to protect against neuron death. Overall, the changes in the expression of category 3 genes suggest the presence of axonal dysfunction and increased pro-apoptotic signaling in *Pink1^−/−^* mice. This in turn may activate regenerative programs, including axonal sprouting and outgrowth, or activation and recruitment of neuronal stem cells that depend on JunB and amphiregulin.

### Altered cytokine expression in the striatum of *Pink1^−/−^* mice after peripheral inflammation

As discussed above, upregulation of genes that antagonize innate immune responses suggests that compensatory gene expression may prevent neuroinflammation in *Pink1*-deficient mice. Consistent with this idea, we found that the expression levels of twelve cytokines measured by ELISA in the striatum were comparable between *Pink1^−/−^* and wildtype mice. However, after peripheral LPS treatment *Pink1^−/−^* mice expressed higher levels of striatal IL-1β, IL-10 and IL-12 than wildtype controls. Although not quite statistically significant, the levels of IL-2, IL-4 and TNF-α also tended to be higher in *Pink1^−/−^* mice. Interestingly, IL-1β, TNF-α, IL-2 and IL-4 levels were shown to be elevated in the brain and cerebrospinal fluid in juvenile Parkinsonism and PD [111], [112], [113]. The same cytokines were also elevated in the serum of patients with idiopathic PD [114], [115]. While higher levels of IL-10 may be protective in PD [115], [116], [117], the pro-inflammatory cytokines IL-1β and TNF-α have been shown to promote and exarcerbate DA neuron death [64], [65], [66], [68], [118], [119]. Additionally, IL-12 may be involved in PD pathogenesis [115]. Unlike wildtype microglia, cultured microglia isolated from neonatal *Pink1^−/−^* mice responded with a significant increase in IL-10 secretion after LPS stimulation, in agreement with elevated levels of IL-10 in *Pink1^−/−^* striatum. Unexpectedly, IL-1β secretion was not potentiated in microglia from either genotype and IL-12 expression was too low to be measured reliably. Nonetheless, the fact that other cytokines, including IL-6, TNF-α and G-CSF were highly induced by the LPS treatment showed that cultured microglia were competent to respond to an inflammatory stimulus. However, the results also suggest that *in vitro* cultured neonatal microglia may not fully mirror the capacity and cytokine profile of adult brain microglia when stimulated with LPS. Although T cells can also synthesize cytokines, we favor the idea that microglial cells are responsible for the increased cytokine expression in the brain, as brain microglial cells strongly respond to LPS via Toll-like receptor activation [63]. In fact, significant up-regulation of various cytokine mRNAs in the striatum was shown to occur as shortly as four hours after peripheral injection of a LPS dose comparable to that used in our studies [120]. In contrast, T cells are present in very low numbers in the brain and part of the antigen-specific adaptive immune response, which takes longer to develop than the short time between LPS injection and cytokine analysis in our experiments (eight hours). Real-time PCR analysis of CD3 expression confirmed that this T cell marker is barely detectable in the striatum and its levels also did not increase after LPS injection. Among the brain resident cells, astrocytes are also known to secrete cytokines, including IL-1β and TNF-α in response to LPS [121]. Our experiments have not addressed a possible role of astrocytes in cytokine production. Future studies with microglia and mixed astrocytes/microglia cultures from the adult brain are needed to determine whether microglia can fully recapitulate the altered cytokine profile observed in the brain after LPS injection, or whether astrocytes may be involved as well. Because peripheral LPS increased the expression of both pro-inflammatory (IL-1β and IL-12) and anti-inflammatory (IL-10) cytokines in *Pink1^−/−^* mice, the net effect on neuronal survival is difficult to predict. However, as *Pink1* defects cause PD in humans, a plausible and testable hypothesis is that abnormal cytokine regulation in the brain of *Pink1^−/−^* mice enhances the vulnerability to inflammation-induced DA neuron death. Interestingly, Parkin-deficient mice treated for 3–6 months with repeated, low doses of systemic LPS developed subtle fine-motor deficits and DA neuron loss that were more pronounced than in wildtype controls [122], indicating that enhanced neuronal vulnerability to inflammation may play a role in recessive Parkinsonism.

### Impaired basal and inflammatory cytokine-induced NF-κB signaling in *Pink1^−/−^* embryonic fibroblasts

The transcription factor NF-κB has many roles in the regulation of cell survival, apoptosis and innate immunity [63]. NF-κB is a key regulator of inflammatory gene expression in microglial cells, which can cause brain inflammation when excessively activated [63]. However, NF-κB is also an important survival factor for neurons and often induces genes favoring survival [123]. For example, NF-κB is required for TNF-α induced expression of the neuroprotective genes Bcl-2 and Bcl-x in primary hippocampal neurons [124] and protects neurons against glucose deprivation, calcium-induced cell death and oxidative stress [125], [126]. Our results show that, compared to wildtype MEF, basal and TNF-α induced NF-κB activity is reduced in fibroblasts derived from *Pink1^−/−^* mice. Moreover, we found that NF-κB activation in response to IL-1β and LPS is also impaired in *Pink1^−/−^* MEF. Taken together, these results show a deficit of *Pink1^−/−^* embryonic fibroblasts in NF-κB signaling induced by cytokines, and suggest that *Pink1* deficiency may predispose neurons to inflammation and oxidative stress-induced apoptosis *in vivo*. Consistent with this, it has recently been shown that *Pink1* enhances NF-κB activation by phosphorylation of Parkin [127]. It should be noted that one group reported that selective inhibition of NF-κB activation reduced MPTP-induced dopaminergic degeneration in the sporadic mouse model of PD [128]. However, another group concluded that NF-κB plays no role in MPTP-induced DA neuron loss [129]. Further studies with *Pink1^−/−^* mice will reveal whether abnormal NF-κB signaling renders these mice more or less susceptible to exogenous stress-induced DA neuron loss.

### Conclusions

In conclusion, our results show that ablation of *Pink1* generates a valuable preclinical model of PD in mice, showing some of the cardinal features of PD including reduced levels of DA and increased DA turnover. These defects are preceded by early transcriptional changes indicative of dopaminergic neuron dysfunction, which may be caused by alterations in mitochondrial Ca^2+^ buffering capacity as well as increased JNK signaling in the substantia nigra. In addition, lack of *Pink1* reduces basal and cytokine-induced NF-κB signaling and increases the levels of both pro-inflammatory and anti-inflammatory cytokines after peripheral LPS challenge. This may lead to decreased neuroprotection against a variety of stresses [123], [126], [130], [131], [132], impaired repair after brain injury [133], [134], reduced response to neurotrophic factors [135] and/or increased vulnerability to inflammation-induced DA neuron loss due to an imbalance between pro-inflammatory and anti-inflammatory mediators [64], [65], [66], [67], [68]. The differential gene expression profile observed in the striatum of *Pink1*-deficient mice is compatible with neuroprotective adaptations to increased MAP kinase signaling and inflammation in the *Pink1^−/−^* brain. Experiments aimed to eliminate or over-express selected genes that are altered in *Pink1^−/−^* mice will ultimately lead to improved animal models for recessive Parkinsonism and the identification of genes and pathways that could serve as targets for future PD therapy.

## Materials and Methods

All animal work has been conducted according to national and international guidelines and has been approved by the Animal Care and Use Committee of the University of Kentucky with identification number 2009-0453. The current approval is valid until 3/9/2011.

### Generation of *Pink1^−/−^* mice

A DNA fragment corresponding to nucleotides 3086-12111 of the mouse *Pink1* locus (Genbank accession NC_000070.5) was amplified by PCR from mouse genomic DNA using a high-fidelity DNA polymerase. The PCR product was subcloned into the vector pGEM-T Easy (Promega) and the entire insert was sequenced and its sequence was found to be identical with the published Genbank entry. Subsequently, a 2011 bp internal fragment spanning *Pink1* nucleotides 7579 (intron 3) to 9590 (intron 5) was excised, removing *Pink1* exons 4 and 5. This fragment was replaced with a PGK-neo-pA cassette amplified by PCR from plasmid ploxPFLPneo (Dr. James Shayman, University of Michigan Medical School). 10^7^ AB2.2 mouse ES cells (129/Sv) were electroporated with 30 µg linearized targeting vector and plated in ES-DMEM/15% ES-qualified FCS at 1−2×10^6^ cells into 10-cm plates containing mitomycin C-inactivated primary mouse embryonic fibroblasts. G418-resistant ES cell colonies were picked individually at day 10 of selection (0.4 mg/ml G418) and clones with a targeted *Pink1* gene were identified by PCR with primer P1 (5′-attgctgaagagcttggcggcgaatgggct-3′) located in the neo gene and primer P2 (5′-tgc tgactgctgcaagagccaggcgatca-3′) located in *Pink1* exon 8 adjacent to the 3′ end of the targeting vector (shown in Figure 1). After confirmation of correct targeting vector insertion at both 5′ and 3′ junctions and ruling out additional random insertions by Southern blot analyses, ES cells from two different clones were injected into C57BL/6 blastocysts. Both clones yielded highly chimeric offspring that were crossed to 129/Sv;C57BL/6 F1 hybrid mice to generate F1 heterozygous mutant mice. The latter were then intercrossed to obtain homozygous mutant and wildtype mice for experiments.

### Southern blots and *Pink1* transcript analysis in mice

10–20 µg genomic DNA from the tails of mice was digested with the indicated restriction enzymes and analyzed by Southern blot using a-P^32^-dCTP labeled random-primed DNA probes (Stratagene Prime-It II kit). Total brain RNA was isolated with the RiboPure kit (Ambion) and converted to cDNA with the Superscript III kit (Invitrogen). Quantitative real-time PCR (Q-PCR) for exon 4-containing transcripts was done with primers 5′-gcgaagccatcttaagcaaa-3′ (exon 3 forward) and 5′-agtagtgtgggggcagcata-3′ (exon 4 reverse). Q-PCR for transcripts encompassing exons 1-3 was done with primers 5′-atccagaggcagttcatggt-3′ (exon 1 forward) and 5′-ttaagatggcttcgctggag-3′ (exon 3 reverse). *Pink1* transcript levels were normalized to 18S rRNA levels detected in the same samples. PCR to detect alternative splicing events was done with forward primers located in exon 3 (5′-ggctggagagtatggagcag-3′ and 5′-agcgaagccatcttaagc aa-3′) and reverse primers located in exon 6 (5′-ccaccacgctctacactgg-3′), exon 7 (5′-caggtatcggct ttgctgta-3′) and exon 8 (5′-ccaggttagccagaaacagc-3′).

### Quantitative gene expression analysis with PCR arrays

Total RNA was isolated from the striatum of mice with the RiboPure kit (Ambion). RNA quality was analyzed on RNA 6000 NanoLab Chips in the Agilent Bioanalyzer 2000 (Agilent Technologies) of the University of Kentucky Microarray Facility. Only RNAs with an RNA integrity number of at least 9 (on a scale of 0–10) were used for analysis. One µg total RNA was converted to cDNA using the RT^2^ first strand synthesis kit (SA Biosciences, MD) and the cDNA reaction was brought to a final volume of 111 µl with H_2_O. 102 µl diluted cDNA reaction was mixed with 2x RT^2^ qPCR Master Mix (SA Biosciences) and H_2_O to reach a final volume of 2.7 ml. 25 µl of the mixture was added into each of the wells of the RT^2^ Profiler PCR Arrays (SA Biosciences) and PCR was carried out on the Roche480 Cycler using a cycling program optimized for PCR arrays (SA Biosciences). Each PCR array contained 84 transcripts of the corresponding signaling pathway, a set of five housekeeping genes as internal controls and additional controls for efficiency of reverse transcription, PCR and the absence of contaminating genomic DNA. Data were analyzed with the ΔΔCt method using the PCR Array Data Analysis Web Portal (SA Biosciences), which includes quality control for each PCR run/array.

### Measurement of mitochondrial Ca^2+^ load capacity and permeability transition

Mitochondria were isolated from whole brains of 8-week old male wildtype or *Pink1^−/−^* mice as described previously [136]. Briefly, brains were homogenized in 2 ml ice-cold isolation buffer (215 mM mannitol, 75 mM sucrose, 0.1% BSA, 20 mM HEPES, 1 mM EGTA, pH 7.2). Homogenates were centrifuged twice at 1,300 *g* for 3 min and then at 13,000 *g* for 10 min at 4°C, using the supernatants and filling up to a final volume of 2 ml with isolation buffer after each centrifugation. The final pellet was resuspended in 0.5 ml isolation buffer and the cells were disrupted in a nitrogen cell disruption bomb (model 4639, Parr Instruments, IL) at 1200 psi for 10 min at 4°C. The resultant crude mitochondrial fraction was placed onto a discontinuous Ficoll gradient (2 ml 7.5% Ficoll solution layered on top of 2 ml 10% Ficoll solution) and centrifuged at 100,000 *g* for 30 min at 4°C. The resulting mitochondrial pellet was carefully separated, resuspended and washed with isolation buffer (without EGTA). After another centrifugation for 10 min at 10,000 *g*, the pellet was resuspended in isolation buffer (without EGTA) and stored on ice until analysis. This method yielded highly coupled mitochondria with RCR of 14.5±2.1 and 12.5±1.9 for wildtype and *Pink1^−/−^* mice (n = 7 preparations). Mitochondrial protein content was measured with the BCA protein assay kit (Pierce, IL). Purified mitochondria (100 µg protein/ml) were placed in constantly stirred, temperature-controlled (37°C) cuvettes in 2 ml respiration buffer (125 mM KCl, 0.1% BSA, 20 mM HEPES, 2 mM MgCl_2_ and 2.5 mM KH_2_PO_4_, pH 7.2), which contained 100 nM calcium green-5N hexapotassium salt (CaG5N) (Molecular Probes, ex506 nm, em532 nm) and 100 nM tetramethylrhodamine (TMRE) (Molecular Probes, ex550 nm, em575 nm) to monitor extra-mitochondrial calcium content and changes in mitochondrial membrane potential (ΔΨ_M_), respectively. Because CaG5N is an indicator of the *extra*-mitochondrial calcium concentration and not imported into mitochondria, data are not confounded by possible differences in the dye loading capacity between wildtype and *Pink1^−/−^* mitochondria. Scans began with a baseline reading followed by addition of mitochondrial substrates (5 mM pyruvate and 2.5 mM malate) at 1 min, 150 mM ADP at 2 min and 1 mM oligomycin at 3 min. At 5 min, calcium chloride (32 mM) was infused gradually through an infusion pump (model 310, KD Scientific, MA) at a rate of 160 nmol per mg protein per minute until the mitochondria were no longer able to sequester calcium, as indicated by a rapid rise in CaG5N signal and loss of ΔΨ_M_. The chemical uncoupler carbonyl cyanide 4-(trifluoromethoxy)phenylhydrazone (FCCP) was added as a positive control at the end of each run. In some experiments the PTP inhibitor CsA (1 µM) was added at the start of the experiment and was shown to increase Ca^2+^ loading capacity. The maximum calcium storage capacity of each mitochondrial preparation was quantified as previously described [25].

### NF-κB activity assay in mouse embryonic fibroblasts

Mouse embryonic fibroblasts (MEF) were prepared from wildtype and *Pink1^−/−^* embryos at 15.5–16.5 dpc as described (Nagy *et al*., 2003: Manipulating the Mouse Embryo, 3^rd^ edition, CSHL Press). Cells were grown in DMEM, 10% fetal bovine serum (FBS) supplemented with penicillin/streptomycin, 0.2 mM L-glutamine and 0.1 mM 2-mercaptoethanol (all from Invitrogen) in a 37°C incubator with 5% CO_2_. For experiments, cells were plated in 24-well plates at 50,000 cells per well and transfected the next day in fresh medium with 800 ng pNF-κB-luc plasmid (Clontech) mixed with 1.6 µl lipofectamine LTX and PLUS reagent prepared in OPTI-MEM (all from Invitrogen). Comparable transfection efficiencies were confirmed with pEGFP-N1 plasmid (Clontech). For each condition wildtype and *Pink1^−/−^* cells were transfected with the same DNA/lipofectamine mixture. 24 hours after transfection, medium was changed and cells were stimulated with 10 ng/ml IL-1β, 30 ng/ml TNF-α (R&D Systems, MN) or 100 ng/ml LPS from *Salmonella minnesota* (Sigma, MO). Eight hours after stimulation cells were lysed in cell culture lysis buffer and luciferase activity measured with the luciferase reporter assay system (Promega, WI). Luminescence was measured in a luminometer (TD-20/20, Turner Design, Promega) and normalized to total protein present in each sample (determined with BCA kit). Comparable transfection efficiency was confirmed by quantification of EGFP-positive cells in several wells of wildtype and *Pink1^−/−^* MEF transfected in parallel in the same experiments, using images taken with an Axiovert 40 fluorescence microscope (Carl Zeiss, Germany) and analyzed with AxioVision software version 4.8 (Carl Zeiss, Germany).

### Expression of phospho-c-Jun and TH in the substantia nigra

Brains from eight-week old wildtype and *Pink1^−/−^* mice were fixed in 4% paraformaldehyde for 48 hrs at 4°C followed by slow immersion in 30% (w/v) sucrose in PBS for 4 days. Thirty µm-thick sections were prepared with a sliding freezing microtome (Microm, Germany) and stored in cryopreservation solution (30% (w/v) sucrose, 1% (w/v) polyvinylpyrrolidone, 30% ethylene glycol, 100 mM sodium phosphate buffer, pH 7.2). Sections containing the substantia nigra were washed in Tris-buffered saline (TBS) and blocked in 5% donkey serum, 0.2% BSA, 0.3% Triton X-100, 0.01% sodium azide in TBS for one hour at room temperature (RT). Sections were incubated overnight at RT with 1∶300 diluted phospho-c-Jun antibody (Cell Signaling, MA) in 2% donkey serum, 0.2% BSA, 0.15% Triton X-100, 0.01% sodium azide. After three washes in TBS the sections were incubated for 1 hour at RT with 1∶500-diluted biotin-SP-conjugated donkey anti-rabbit IgG (Jackson Immunoresearch) in 2% donkey serum, 0.2% BSA in TBS. After washing as above sections were incubated in freshly prepared, filtered nickel-diaminobenzidine (nickel-DAB) solution (1.5g nickel ammonium sulfate, 25 mg DAB in 400 ml of 0.1 M sodium acetate buffer). Prior to use 2 µl of 30% hydrogen peroxide was added to the nickel-DAB solution. Color development in the sections (placed on a shaker) was monitored and the reaction was stopped before background developed in control sections incubated with secondary antibody only. Subsequently, the same sections were used to identify TH-expressing neurons by fluorescent IHC. For this purpose sections were washed in TBS, re-blocked for 20 min in blocking buffer and incubated overnight at 4°C with 1∶1500 diluted sheep anti-TH antibody (Pel-Freeze, AR). 1∶500 diluted DyLight549-conjugated anti-sheep IgG (Invitrogen) was used for 1 hour at RT as the secondary antibody. After extensive washing in TBS, sections were mounted with Permount (Fisher Scientific, NJ) and images captured through phase-contrast (phospho-c-Jun) or fluorescent filters (TH) using an Axiovert 40 microscope equipped with an AxioCam MRc5 digital camera and AxioVision software (Carl-Zeiss, Germany).

### Measurement of cytokines in the striatum and medium of isolated cultured microglia cells

Six month-old mice were left untreated or given intra-peritoneal (i.p.) injections of 0.33 µg LPS from *Salmonella minnesota* per gram body weight. Eight hours after injection mice were euthanized and brains harvested. Dose of LPS and time of analysis were chosen based on a previous publication [120]. The striatum was dissected from each brain on ice and homogenized with T-PER tissue protein extraction reagent (Pierce Biotechnology, IL) containing protease inhibitor cocktail (Sigma, MO) (20 ml buffer/mg tissue). Homogenates were centrifuged (10,000 g, 5 min, 4°C) and supernatants collected and stored at −80°C until analysis. Cytokines in the striatal lysates (100 µg total protein) were measured using Multi-Analyte ELISArray plates (SA Biosciences, MD) according to the manufacturer's instructions. The plates allow for the simultaneous relative quantification of IL-1α, IL-1β, IL-2, IL-4, IL-6, IL-10, IL-12, IL-17α, interferon-γ, TNF-α, G-CSF and GM-CSF. Primary microglia cultures were prepared from the cerebral cortices of neonatal (1 day-old) wildtype and *Pink1^−/−^* mice as previously described for rats [137]. Briefly, forebrains were cleared from meninges, minced and gently dissociated by repeated pipetting in PBS followed by filtration through a 70-µm cell strainer (Falcon). Cells were collected by centrifugation (1000 rpm, 10 min), resuspended in DMEM containing 10% fetal calf serum and antibiotics (40 U/ml penicillin and 40 µg/ml streptomycin), and cultured on 10-cm cell culture dishes (1.3×10^5^ cells/cm^2^) in 5% CO_2_ at 37°C. Floating microglia, collected by shaking cultures at 90 rpm for 5 min, were harvested every week (between 2–7 weeks) and re-seeded into wells of a 24-well plate to give pure microglial cultures. For experiments, cultures were washed to remove non-adherent cells and fresh medium was added. Cells (n = 8 wells/condition) were treated with 100 ng/ml LPS (Sigma) or left untreated for 24 h. Supernatants were collected, clarified by centrifugation and used to quantify cytokines as described above.

### Quantification of striatal catecholamines and nigral dopaminergic neurons

Striatal tissue was dissected on an-ice-cold plate, tissue pieces weighed and snap-frozen in liquid nitrogen. Samples were homogenized in 300 µl of ice-cold 0.1 M perchloric acid containing the internal standard dihydroxybenzylamine and centrifuged for 5 min at 15,000× *g*. Supernatants were filtered through a 0.22 µm pore size membrane and diluted with HPLC mobile phase prior to injection onto the HPLC column. Striatal tissue levels of DA, 3, 4-dihydroxyphenylacetic acid (DOPAC) and homovanillic acid (HVA) were measured by reverse phase HPLC coupled to electrochemical detection as described [138]. TH-positive neurons in 30 µm-thick sections of the substantia nigra were stained with a sheep anti-TH antibody (Pel-Freeze) and detected with biotinylated secondary antibody and the DAB method as described by us previously [139], [140]. The optical fractionator [141], [142] and dissector method for unbiased stereological cell counting was used to estimate the numbers of dopaminergic neurons using the Stereologer System and software (Stereology Research Center, www.disector.com). Five wildtype and six *Pink1^−/−^* mice were analyzed and neurons in every fourth section of the SNc were counted (12 sections per mouse, both hemispheres).

### Data analysis and statistics

Data were analyzed using the Student's t-test. Results are expressed as mean ± standard deviation. PCR Array data were analyzed using the ΔΔC_t_ method and the web tools of SA Biosciences (http://www.sabiosciences.com/pcr/arrayanalysis.php).
